# A Possible Link between Gut Microbiome Composition and Cardiovascular Comorbidities in Psoriatic Patients

**DOI:** 10.3390/jpm12071118

**Published:** 2022-07-09

**Authors:** Virginia Valentini, Valentina Silvestri, Agostino Bucalo, Federica Marraffa, Maria Risicato, Sara Grassi, Giovanni Pellacani, Laura Ottini, Antonio Giovanni Richetta

**Affiliations:** 1 Department of Molecular Medicine, Sapienza University of Rome, 00161 Rome, Italy; virginia.valentini@uniroma1.it (V.V.); valentina.silvestri@uniroma1.it (V.S.); agostino.bucalo@uniroma1.it (A.B.); 2 Unit of Dermatology, Department of Internal Medicine and Medical Specialties Sapienza, University of Rome, 00161 Rome, Italy; femarraffa@gmail.com (F.M.); maria.risicato@icloud.com (M.R.); sara.grassi@uniroma1.it (S.G.); giovanni.pellacani@uniroma1.it (G.P.); antoniorichetta@hotmail.com (A.G.R.)

**Keywords:** psoriasis, comorbidities, cardiovascular disease, gut microbiome, biologic therapy, 16S rRNA gene sequencing

## Abstract

Cardiovascular disease (CVD) is one of the most common comorbidities that may affect psoriatic patients. Several exogenous and endogenous factors are involved in the etiology and progression of both psoriasis and CVD. A potential genetic link between the two diseases has emerged; however, some gaps remain in the understanding of the CVD prevalence in psoriatic patients. Recently, the role of the gut microbiome dysbiosis was documented in the development and maintenance of both diseases. To investigate whether gut microbiome dysbiosis might influence the occurrence of CVD in psoriatic patients, 16S rRNA gene sequencing was performed to characterize the gut microbiome of 28 psoriatic patients, including 17 patients with and 11 without CVD. The comparison of the gut microbiome composition between patients with and without CVD showed a higher prevalence of *Barnesiellaceae* and *Phascolarctobacterium* in patients with CVD. Among patients with CVD, those undergoing biologic therapy had lower abundance levels of *Barnesiellaceae,* comparable to those found in patients without CVD. Overall, these findings suggest that the co-occurrence of psoriasis and CVD might be linked to gut microbiome dysbiosis and that therapeutic strategies could help to restore the intestinal symbiosis, potentially improving the clinical management of psoriasis and its associated comorbidities.

## 1. Introduction

Psoriasis is a chronic inflammatory and immune-mediated disease characterized by cutaneous and systemic manifestations, and affecting 1–3% of the population in Western Europe [1]. Compared with the general population, psoriatic patients have an increased risk of developing other chronic diseases as comorbidities, including cardiovascular diseases (CVDs). CVDs are one of the most common comorbidities in psoriatic patients, and this may affect the severity of the disease and decrease both the quality of life and the lifespan of psoriatic patients [2].

A combination of several exogenous and endogenous factors, including genetic predisposition, is well documented in the etiology and progression of both psoriasis and CVD [3,4,5,6,7]. The presence of systemic inflammation in combination with metabolic abnormalities might explain the high incidence of CVD in psoriatic patients [2,8].

Genetic and genome-wide association studies have recently allowed for the identification of a common genetic background of psoriasis and concomitant cardiovascular risk factors, suggesting pleiotropic mechanisms of interactions involving pro-inflammatory pathways [6]. However, other factors besides genetic background may explain the prevalence of CVD in psoriasis patients.

Gut microbiome dysbiosis is considered as a triggering factor for psoriasis onset as it may promote chronic systemic inflammation. Dysbiosis may indeed contribute to the increase in bacteria promoting the production of harmful compounds involved in the inflammatory response, with a central role in the systemic inflammation of several diseases [9,10].

Significant alterations of the gut microbiome composition were found in psoriatic patients compared with healthy controls, mainly including a reduction in *Bacteroidetes* and increase in *Firmicutes* phyla [9].

Cumulative evidence has also demonstrated that the gut microbiome may have a role in the development and maintenance of CVD [11,12]. Compared with healthy controls, patients with CVD showed a peculiar gut microbiome profile, including a reduction in *Bacteroidetes* and increase in *Proteobacteria* phyla [13].

Despite the established role of the gut microbiome composition in the development and maintenance of both psoriasis and CVD, a possible link between the gut microbiome composition and the occurrence of CVD in psoriatic patients is still unknown.

In this study, we characterized and compared the gut microbiome composition in psoriatic patients with and without CVD to investigate whether gut dysbiosis might influence the occurrence of CVD in psoriatic patients, with possible clinical relevance.

## 2. Materials and Methods

A cohort of 28 psoriatic patients, including 17 cases with CVD and 11 cases without CVD, were diagnosed and enrolled at the Psoriasis Center of Policlinico “Umberto I”, Sapienza University of Rome. All patients involved in this study signed an informed consent form with a detailed description of the study protocol, approved by the local ethics committee of Sapienza University of Rome (protocol 873/13). The study was performed according to the principles of the Declaration of Helsinki.

The main personal and clinical-pathological data including sex, age at enrollment and at diagnosis, duration of the disease, body mass index (BMI), type and degree of psoriasis, and anti-psoriatic biological treatment were collected for all patients.

For each case, fecal samples were obtained within 24 h of production and were stored at −20 °C in sterile containers until use. To reduce possible biases due to environmental effects on the microbiome composition, the study enrolled Caucasian individuals from the same geographic area (Lazio, Italy), all of whom had a similar diet and had not received probiotics or antibiotics in the 6 months prior to recruitment. Fecal bacterial DNA was extracted using the commercially available InviMag Stool DNA Kit/KF96 (Invitek Molecular, Berlin, Germany) and analyzed by 16s rRNA gene sequencing on the Illumina MiSeq platform. The sequencing data were processed using QIIME2 version 2019.1 and DADA2 software, and the details including raw and clean data per sample and number of reads passing quality filters were as previously reported [10]. The operational taxonomic units (OTUs) per sample were generated after the filtering by quality and length and purification from chimeric sequences; the rarefaction analysis was performed on the OTU table in order to normalize samples at the minimum number of reads, as previously described [10]. The sequencing data are available upon reasonable request to the corresponding author.

Bioinformatics analyses were performed to determine the diversity in the gut microbiome composition of psoriatic patients with and without CVD.

Taxonomy was assigned using the Greengenes v.13-8 database and the differential microbial abundance between the two groups was performed using the DeSeq2 package of R/Bioconductor, as previously described [10].

Statistical analysis of the clinical-pathological features in the case series was performed using the Wilcoxon rank-sum test for numerical variables, and the χ^2^ test for categorical variables. Results were considered statistically significant at *p* ≤ 0.05.

## 3. Results and Discussion

The personal and clinical-pathological data of the 28 psoriatic patients included in the study are shown in Table 1.

**Figure 1:**
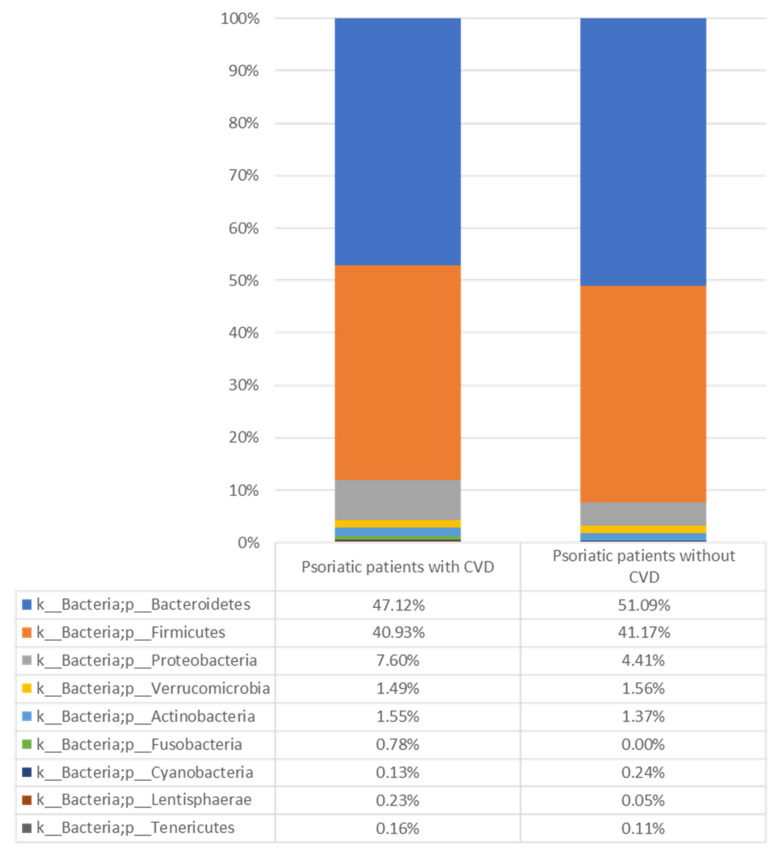
Relative abundance (%) of the gut microbes identified at phylum level in psoriatic patients with and without CVD. CVD: cardiovascular disease.

Psoriatic patients with CVD were significantly older at enrollment (*p* = 0.001) and at diagnosis (*p* = 0.049) and had a significantly higher BMI (*p* = 0.003) compared with psoriatic patients without CVD. Among psoriatic patients with and without CVD, five and four cases, respectively, were undergoing biological therapy. We subsequently examined the gut microbiome composition of psoriatic patients with and without CVD, analyzing the abundance of specific bacterial groups at various taxonomic levels. At the phylum level, we observed a significantly higher abundance of *Proteobacteria*, known to promote a status of systemic inflammation [13] in psoriatic patients with CVD, compared with those without CVD (*p* = 0.045) (Figure 1 and Supplementary Figure S1).

Notably, an increased abundance of Proteobacteria was previously reported in patients with coronary artery disease [13]. Our findings suggest that a higher abundance of Proteobacteria in psoriatic patients with CVD might contribute to inflammatory processes related to both psoriasis and CVD by means of increasing the production of inflammatory cytokines. In addition, we observed a significantly higher proportion of the family *Barnesiellaceae*, the genera *Coprococcus* and *Phascolarctobacterium*, and the species *Bacteroides ovatus* in psoriatic patients with CVD (Table 2).

A higher abundance of *Barnesiellaceae* and *Phascolarctobacterium*, known to produce elevated quantities of short-chain fatty acids associated with both hypercholesterolemia and hypertriglyceridemia, was previously reported in CVD patients compared with healthy controls [12,14]. Our findings suggest that a higher prevalence of *Barnesiellaceae* and *Phascolarctobacterium* might be considered as risk factors for developing CVD in psoriatic patients.

Previous studies showed controversial associations of *Barnesiellaceae* and *Phascolarctobacterium* with metabolic status (measured as BMI), as some studies reported higher levels in higher BMI patients, and others reported an inverse correlation [15,16,17,18]. These contrasting results may be due to other additional features that may act as confounding factors. In our series, psoriatic patients with CVD were significantly older and had a significantly higher BMI compared with psoriatic patients without CVD. Given the relatively small number of patients analyzed, we cannot rule out the possible effects of age and BMI as confounding factors for the observed associations. On the other hand, our observations may hint at a possible link between gut microbiome composition and metabolic factors, for example, a high BMI, suggesting a possible concurrent role of gut microbiome alterations and traditional cardiovascular risk factors in psoriatic patients. 

**Figure 2:**
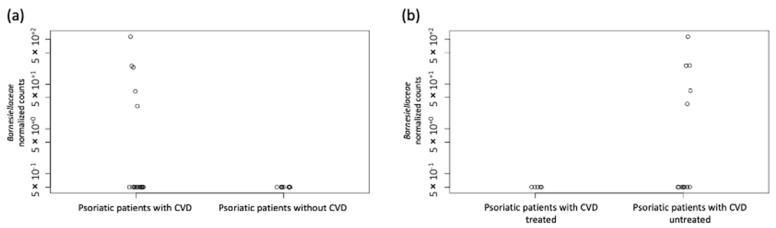
The abundance of the family Barnesiellaceae in psoriatic patients analyzed in this study. (**a**) Levels of the family Barnesiellaceae in the gut microbiome of psoriatic patients with and without CVD. (**b**) Levels of the family Barnesiellaceae in the gut microbiome of psoriatic patients with CVD treated and untreated with biological therapy. Each circle in the figure represents a psoriatic patient. CVD: cardiovascular disease.

Among psoriatic patients with CVD, patients undergoing biological therapy had a lower abundance of the family *Barnesiellaceae* (*p* = 7.79 × 10^−11^) and showed abundance levels comparable to those found in psoriatic patients without CVD (Figure 2a,b).

Whether biological treatment may affect the gut microbiome, with a possible impact on cardiovascular comorbidities in psoriatic patients, deserves to be further validated in a larger cohort of patients.

The order *Clostridiales* and the species *Alistipes finegoldii* and *Roseburia faecis* were found significantly more abundant in psoriatic patients without CVD (Table 2). *Alistipes finegoldii* and *Roseburia faecis* were suggested as playing a role in improving obesity [12] and in producing butyrate with vasodilatory properties [14], respectively. Our findings suggest that these bacteria might positively impact traditional cardiovascular risk factors in psoriatic patients.

This study has a few limitations. First, it is a pilot study on a small cohort of patients; thus, it was not possible to exclude the possible influence of confounding factors for the observed associations. On the other hand, the analyzed cohort was collected through stringent inclusion criteria in order to minimize environmental biases. Another possible limitation is represented by data analysis. It is well known that microbiome data analysis is a computational challenge, and a standardization of the best pipeline is currently lacking. The analysis pipeline, whose results are shown here, is based on evidence from previous results and evidence from recent methodological comparisons [10,19]. 

Further studies using multiple functional and computational approaches are needed to confirm our observations regarding the gut microbiome dysbiosis and the possible functional relationship among dysregulated taxa in the risk of CVD in psoriatic patients.

## 4. Conclusions

This study highlighted differences in the gut microbiome composition in psoriatic patients with and without CVD and suggested that gut microbiome dysbiosis may be associated with an increased risk of CVD in psoriatic patients, in addition to traditional cardiovascular risk factors (i.e., high BMI), with possible clinical implications in the management of psoriasis and its associated comorbidities. Furthermore, our findings hinted at a possible impact of biological therapy on the gut microbiome composition, an observation which deserves further validation. Thus, preventive and therapeutic strategies aimed at restoring the gut microbiome symbiosis might potentially represent useful approaches in the management of psoriasis and its associated comorbidities. Further studies on larger cohorts of patients will provide more insights regarding the impact of biological drugs on gut microbiome composition and on bacteria that could play an important role for the host’s cardiovascular health, in terms of treatment and prevention of associated diseases. 

## Figures and Tables

**Table 1:** Clinical-pathological features of the 28 psoriatic patients included in the study.

Clinical-Pathological Features	Total Patients N = 28 (%)	Patients with CVD N = 17 (%)	Patients without CVD N = 11 (%)	*p*-Value ^1^
**Sex**				
Male	19 (67.8%)	11 (80.0%)	8 (72.7%)	
Female	9 (32.2%)	6 (20.0%)	3 (27.3%)	0.197
**Age at enrollment** (mean ± standard error)	59.7 (±2.7)	67.1 (±1.8)	48.9 (±4.5)	**0.001**
**Age at diagnosis** (mean ± standard error)	31.7 (±3.1)	37.2 (±4.2)	23.6 (±3.5)	**0.049**
**Duration of the disease** (mean ± standard error)	21.1 years (±2.6)	22.0 years (±3.9)	19.9 years (±3.0)	1.000
**BMI** (mean ± standard error)	28.2 (±0.9)	30.1 (±1.1)	25.3 (±1.1)	**0.003**
**Type of psoriasis**				
Plaque	24 (85.8%)	16 (94.1%)	8 (72.7%)	
Guttate	2 (7.1%)	1 (5.9%)	1 (9.1%)	
Arthropathic	2 (7.1%)	0 (0.0%)	2 (18.2%)	0.170
**Degree of psoriasis**				
Mild	3 (10.7%)	1 (5.9%)	2 (18.2%)	
Moderate	10 (35.7%)	6 (35.3%)	4 (36.4%)	
Severe	15 (53.6%)	10 (58.8%)	5 (45.4%)	0.557
**Biological treatment**				
Treated ^2^	9 (32.1%)	5 (29.4%)	4 (36.4%)	
Untreated	19 (67.9%)	12 (70.6%)	7 (63.6%)	0.700

CVD: cardiovascular disease. BMI: body mass index. **^1^** From Wilcoxon rank-sum test for numerical variables, and χ^2^ test for categorical variables. *p*-value < 0.05 in bold text. **^2^** Three out of five patients with CVD and two out of four patients without CVD were in treatment with an anti-tumor necrosis factor-α drug (golimumab or adalimumab), whereas two patients in both groups were in treatment with a drug targeting both IL-12 and IL-23 (ustekinumab).

**Table 2:** List of the significantly differentially abundant taxonomic groups in psoriatic patients with and without CVD.

Taxonomic Group	Abundance Status	log2 Fold Change	Adjusted *p*-Value
p__Bacteroidetes; c__Bacteroidia; o__Bacteroidales; f__[Barnesiellaceae]	Higher in psoriatic patients with CVD	−23.34886701	1.05 × 10^−12^
p__Firmicutes; c__Clostridia; o__Clostridiales; f__Lachnospiraceae; g__Coprococcus	Higher in psoriatic patients with CVD	−22.4934482	7.37 × 10^−12^
p__Firmicutes; c__Clostridia; o__Clostridiales; f__Veillonellaceae; g__Phascolarctobacterium	Higher in psoriatic patients with CVD	−23.97162303	2.65 × 10^−15^
p__Bacteroidetes; c__Bacteroidia; o__Bacteroidales; f__Bacteroidaceae; g__Bacteroides; s__ovatus	Higher in psoriatic patients with CVD	−22.82969217	5.42 × 10^−13^
p__Firmicutes; c__Clostridia; o__Clostridiales	Higher in psoriatic patients without CVD	23.26865584	9.73 × 10^−13^
p__Bacteroidetes; c__Bacteroidia; o__Bacteroidales; f__Rikenellaceae; g__Alistipes; s__finegoldii	Higher in psoriatic patients without CVD	23.55698273	5.42 × 10^−13^
p__Firmicutes; c__Clostridia; o__Clostridiales; f__Lachnospiraceae; g__Roseburia; s__faecis	Higher in psoriatic patients without CVD	23.62161748	5.42 × 10^−13^

CVD: cardiovascular disease.

## Data Availability

The data that support the findings of this study are available from the corresponding author upon reasonable request.

## Supplementary Materials

The following supporting information can be downloaded at: https://www.mdpi.com/article/10.3390/jpm12071118/s1, Figure S1: Relative abundance (%) of the gut microbes identified at phylum level represented by stacked bars of each subject (1–28) from each group of psoriatic patients with and without CVD (1–17 and 18–28, respectively).
