# Supplementary material for: A Possible Link between Gut Microbiome Composition and Cardiovascular Comorbidities in Psoriatic Patients
Source: J Pers Med. 2022 Jul 9;12(7):1118. doi: 10.3390/jpm12071118 (PMC9324618; doi:10.3390/jpm12071118)
Supplement: Supplementary file 1 [file jpm-12-01118-s001.zip › jpm-1742766-supplementary.pdf]

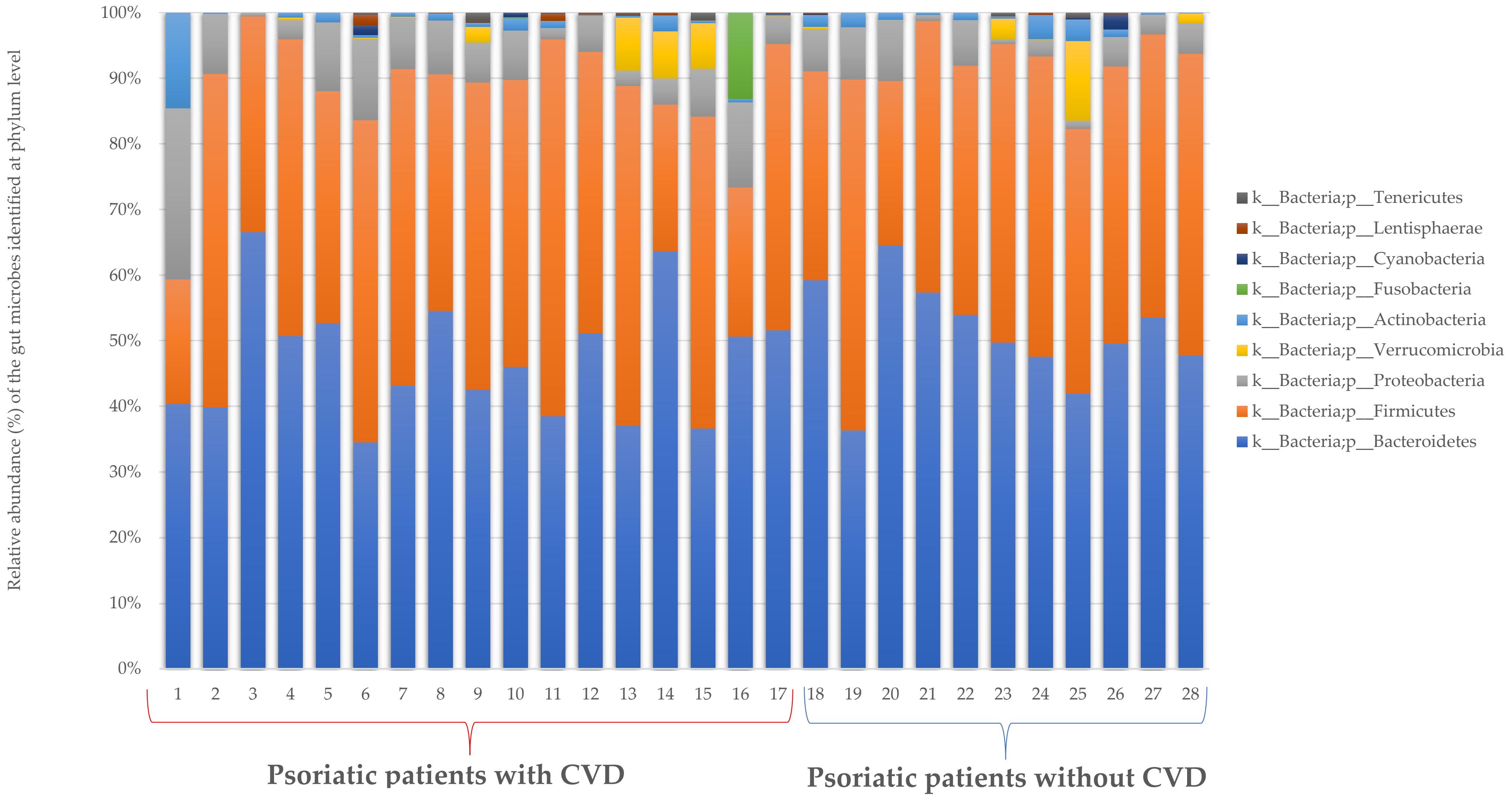

Figure S1. Relative abundance (%) of the gut microbes identified at phylum level represented by stacked bars of each subject (1-28) from each group of psoriatic patients with and without CVD (1-17 and 18-28, respectively). CVD: cardiovascular disease.
